# Two Cases of Lung Cancer-Associated Horner Syndrome With Divergent Outcomes Following Radiotherapy

**DOI:** 10.7759/cureus.111788

**Published:** 2026-06-30

**Authors:** Takuya Sasaki, Yojiro Ishikawa, Satoshi Teramura, Yasuhiro Nakamura, Takayuki Yamada

**Affiliations:** 1 Medical Education, Tohoku Medical and Pharmaceutical University, Sendai, JPN; 2 Radiology, Tohoku Medical and Pharmaceutical University, Sendai, JPN; 3 Pathology, Tohoku Medical and Pharmaceutical University, Sendai, JPN

**Keywords:** horner syndrome, lung cancer, palliative radiotherapy, pancoast tumour, ptosis

## Abstract

Horner syndrome is a well-recognized manifestation of sympathetic pathway disruption in lung cancer, particularly in apical lesions; however, its reversibility after treatment varies and remains difficult to predict. We report two cases of lung cancer-associated Horner syndrome that showed contrasting neurological outcomes following radiotherapy. In the first case, contrast-enhanced computed tomography (CT) demonstrated an apical lung tumor with a close anatomical relationship to adjacent neurovascular structures, and Horner syndrome failed to show sustained improvement after definitive radiotherapy. In the second case, contrast-enhanced CT revealed a supraclavicular lymph node metastasis without radiological evidence of direct neural invasion, and the neurological symptoms improved and remained stable after palliative radiotherapy. The differing post-treatment courses observed in these cases suggest that pre-treatment imaging findings, particularly the anatomical relationship between tumor lesions and surrounding neurovascular structures, may assist in prognostic estimation and patient counseling regarding the potential reversibility of Horner syndrome in patients with lung cancer.

## Introduction

Horner syndrome is a neurological syndrome characterized by the classic triad of miosis, ptosis, and facial anhidrosis, resulting from disruption of the sympathetic pathway extending from the hypothalamus to the eye [1]. In lung cancer, it most commonly arises from invasion or compression of the cervicothoracic sympathetic trunk by apical tumors (Pancoast tumors) or metastatic involvement of mediastinal or supraclavicular lymph nodes. Although Pancoast tumors account for only 3%-5% of lung cancers, the presence of Horner syndrome is generally regarded as a clinical indicator of advanced local tumor progression [2,3]. Horner syndrome secondary to non-apical primary lung cancer is considerably less common and has been described mainly in isolated case reports. In such cases, cervical or supraclavicular lymph node metastases may compress the sympathetic pathway, indicating that metastatic lesions can also serve as causative mechanisms [4,5]. Despite these reports, the overall clinical characteristics and prognostic implications of Horner syndrome in non-apical lung cancer remain insufficiently characterized. Traditionally, neoplasm-associated Horner syndrome has been considered to have limited reversibility, with neurological symptoms frequently persisting even after treatment of the underlying malignancy [1]. However, sporadic reports have described partial or complete symptom improvement following tumor control [6,7], suggesting that reversibility may depend on the underlying mechanism of sympathetic pathway injury. Importantly, the extent to which pre-treatment imaging findings can predict neurological recovery has not been clearly established. In particular, whether radiologic differentiation between direct neural invasion and compressive involvement influences symptom reversibility remains unclear. In this report, we present two cases of lung cancer-associated Horner syndrome demonstrating divergent clinical courses following radiotherapy. By comparing pre-treatment imaging findings regarding the spatial relationship between the tumor and the sympathetic pathway with post-treatment neurological outcomes, we explore factors that may influence symptom evolution and potential reversibility.

## Case presentation

Case 1

A man in his 40s presented with chief complaints of cough, hoarseness, and dysphagia. These symptoms had emerged approximately three weeks before presentation, prompting him to visit a local otolaryngology clinic, where right lower cervical lymphadenopathy was identified. He was subsequently referred for further evaluation.

Contrast-enhanced chest computed tomography (CT) revealed a 55-mm irregular mass in the right lung apex with mediastinal extension and suspected involvement of adjacent major vessels (Figure 1). Comprehensive staging evaluation, including brain MRI, showed no evidence of distant metastasis. 

**Figure 1:**
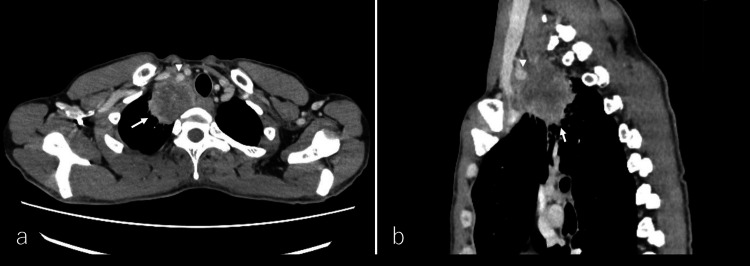
Chest CECT (contrast-enhanced computed tomography) findings of Case 1: Pancoast-type lung adenocarcinoma. (a) Axial image; (b) sagittal reconstruction image An irregularly marginated tumor lesion with a maximum diameter of 55 mm is identified in the right lung apex (white arrows in a and b). The lesion circumferentially encases the right subclavian artery over more than 180 degrees (white arrowhead in b). Additionally, the tumor is in contact with the brachiocephalic artery, superior vena cava, trachea, and esophagus, demonstrating typical features of a Pancoast tumor. No bone destruction or definitive lymphadenopathy is observed. No intrapulmonary metastasis, pathologic lymphadenopathy, or bone destruction was observed on CECT. CECT, contrast-enhanced computed tomography.

On pathologic examination, atypical cells characterized by enlarged, irregular nuclei and pale eosinophilic cytoplasm exhibited infiltrative growth in variably sized solid nests. Immunohistochemical staining revealed positivity for TTF-1 in the tumor cells (Figure 2). Molecular analysis demonstrated PD-L1 expression of 100% and a positive KRAS G12C mutation. 

**Figure 2:**
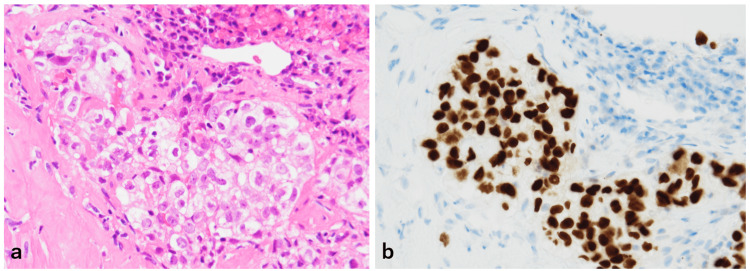
Histopathological and immunohistochemical findings of Case 1. (a) Hematoxylin and eosin staining; (b) TTF-1 immunohistochemical staining (a) Hematoxylin and eosin (H&E) staining shows atypical tumor cells with enlarged, irregular nuclei and pale eosinophilic cytoplasm forming variably sized solid nests with infiltrative growth into the fibrous stroma. (b) Immunohistochemical staining demonstrates nuclear positivity for thyroid transcription factor-1 (TTF-1) in the tumor cells, supporting the diagnosis of primary lung adenocarcinoma.

Based on these findings, the patient was diagnosed with Pancoast-type apical lung cancer with a clinical stage of cT4N0M0, Stage IIIA. Definitive concurrent chemoradiotherapy with carboplatin and nab-paclitaxel was selected as the treatment strategy. Radiotherapy was delivered at a total dose of 60 Gy in 30 fractions using standard target delineation and motion assessment (Figure 3). 

**Figure 3:**
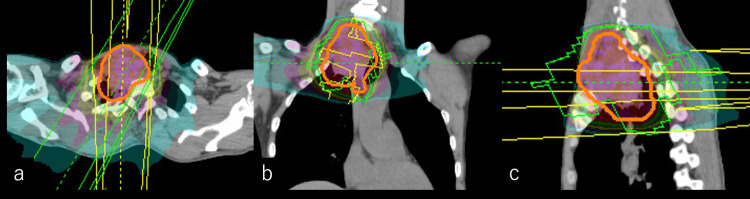
Radiation therapy planning for a right apical lung tumor showing the irradiation fields on (a) axial, (b) coronal, and (c) sagittal images The right apical lung tumor was defined as the gross tumor volume (GTV). A 0.5-cm margin was added to the GTV to generate the clinical target volume (CTV), and an additional 0.5-cm margin was applied to create the planning target volume (PTV). The CTV is depicted as the orange region of interest (ROI). Given the apical location of the lesion, tumor motion was assessed using four-dimensional computed tomography (4D-CT), which demonstrated respiratory motion of ≤5 mm. Based on these findings, definitive radiotherapy with a total dose of 60 Gy delivered in 30 fractions was planned and administered.

Following treatment initiation, tumor shrinkage was observed at a cumulative dose of approximately 40 Gy, accompanied by evident improvement of the right-sided ptosis (Figure 4b). However, immediately after completion of radiotherapy, the right-sided ptosis recurred and deteriorated to nearly the same severity as observed at baseline (Figure 4c). Treatment-related adverse events included Grade 1 radiation dermatitis (mild erythema) and Grade 2 radiation esophagitis (sensation of obstruction), both of which were manageable with supportive care. At more than six months post-radiotherapy, while tumor shrinkage was maintained, no improvement in ptosis was observed. 

**Figure 4:**
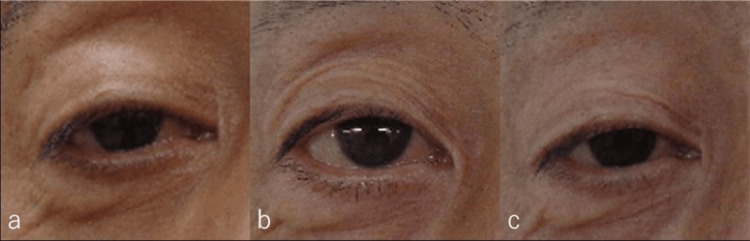
Treatment course and changes in Horner syndrome in Case 1. (a) Baseline; (b) at cumulative dose of 40 Gy; (c) at treatment completion Right-sided ptosis due to Horner syndrome was observed at baseline. Following definitive concurrent chemoradiotherapy (60 Gy in 30 fractions), improvement of ptosis was noted at a cumulative dose of 40 Gy concurrent with tumor shrinkage; however, immediately after completion of radiotherapy, the ptosis recurred and deteriorated to nearly the same severity as observed at baseline.

Case 2

A man in his 70s presented with a chief complaint of cough. Approximately two months before presentation, he developed a persistent cough and visited a local clinic. Laryngoscopic examination revealed no abnormalities, and antitussive medication was prescribed with continued observation; however, no symptomatic improvement was achieved. He subsequently presented to a general hospital, where right supraclavicular lymphadenopathy was identified, prompting referral to the Department of Otolaryngology at our institution for further evaluation. Chest CT demonstrated enlarged right supraclavicular lymph nodes (Figure 5). In addition, a 16-mm irregularly marginated nodule was identified in the right upper lobe (Figure 6). Ultrasound-guided fine-needle aspiration cytology of the lymph node confirmed adequate specimen acquisition; however, no malignant cells were identified, and the sample was deemed nondiagnostic. Although pathologic confirmation of pulmonary origin was not obtained, the presence of an irregular right upper lobe nodule in conjunction with supraclavicular lymphadenopathy raised a strong suspicion of primary lung cancer. Based on the clinical course and imaging findings, the lesion was considered clinically highly suggestive of right upper lobe lung cancer. The patient declined further diagnostic procedures, including bronchoscopy and biopsy, and was subsequently followed by the palliative care department. 

**Figure 5:**
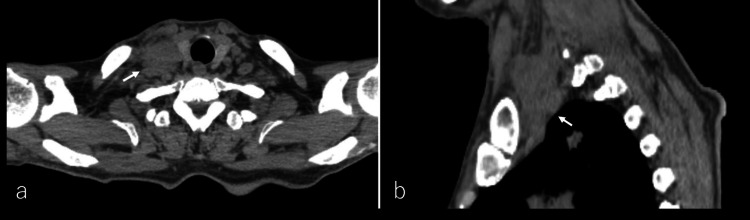
Chest CT findings of Case 2: supraclavicular lymph node metastasis. (a) Axial image; (b) sagittal reconstruction image Enlarged lymph nodes are identified in the right supraclavicular fossa (white arrow). The nodes are located in proximity to the course of the cervical sympathetic trunk; however, no definitive findings suggestive of neural or vascular invasion by the tumor are observed.

**Figure 6:**
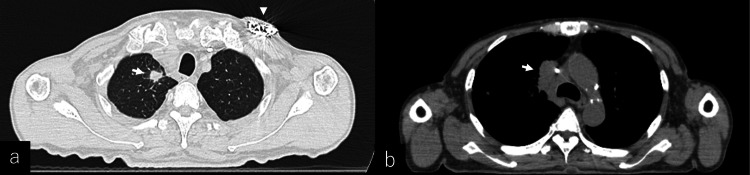
Non-contrast CT showing the presumed primary lesion in the right upper lobe. (a) Lung window; (b) mediastinal window Axial non-contrast chest CT images at different slice levels (a,b) demonstrate a 16 mm irregularly marginated nodule in the right upper lobe (white arrowhead). The lesion depicted in both images represents the same nodule. The arrowhead in (a) indicates the cardiac pacemaker generator and is unrelated to the pulmonary lesion. Based on the radiological findings, this nodule was considered highly suspicious for primary lung cancer.

Approximately six months after the initial consultation, pain corresponding to the right supraclavicular mass developed, accompanied by symptoms of Horner syndrome, including right-sided ptosis and miosis. The patient was referred to our department for pain relief related to the right supraclavicular lymph node and improvement of Horner syndrome.

His medical history included prior cardiac pacemaker implantation and insomnia, as well as a history of suspected sarcoidosis, although details were unclear. He had a smoking history of 15 cigarettes per day for 50 years and no history of alcohol consumption. Physical examination at presentation revealed right-sided ptosis, miosis, and ipsilateral lacrimation, with a tender mass palpable in the right supraclavicular fossa. The enlarged lymph nodes were located in proximity to the course of the cervical sympathetic trunk; however, no definitive findings suggestive of significant neural or vascular invasion were identified.

Palliative radiotherapy was selected as the treatment strategy. Based on the treatment plan and target delineation, palliative irradiation was delivered using 6 MV X-rays to a total dose of 20 Gy in five fractions (Figure 7). 

**Figure 7:**
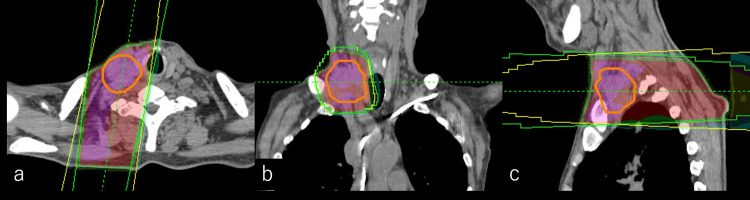
Radiation therapy planning for palliative treatment of a right supraclavicular lymph node metastasis showing the irradiation fields on (a) axial, (b) coronal, and (c) sagittal images The enlarged right supraclavicular lymph node was defined as the gross tumor volume (GTV). A 0.5 cm margin was added to the GTV to generate the clinical target volume (CTV), and an additional 1.0 cm margin was applied to create the planning target volume (PTV). The CTV is represented by the orange region of interest (ROI). Palliative radiotherapy was planned and delivered using 6 MV X-rays, with a total dose of 20 Gy administered in five fractions.

Following completion of radiotherapy, evident improvement of the right-sided ptosis was observed (Figure 8b), and pain in the right supraclavicular fossa was alleviated, resulting in improved quality of life. The patient subsequently declined further oncologic treatment and continued follow-up with the palliative care department. No recurrence of ptosis was documented at the time of the last clinical evaluation. However, detailed long-term neurological follow-up data were not available. 

**Figure 8:**
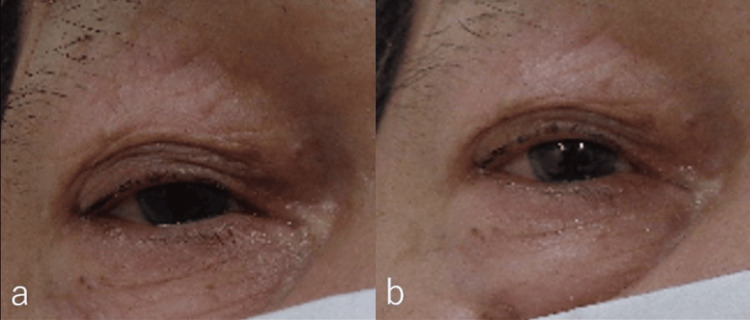
Changes in symptoms before and after radiotherapy in Case 2. (a) Baseline; (b) at treatment completion Palliative radiotherapy (20 Gy in 5 fractions) was administered to the right supraclavicular lymph node. At treatment completion, evident improvement of the right-sided ptosis was observed, with no recurrence noted during follow-up.

## Discussion

In this report, we presented two cases of lung cancer-associated Horner syndrome that demonstrated divergent neurological courses following radiotherapy. In Case 1, despite definitive-dose radiotherapy (60 Gy in 30 fractions), symptom improvement was transient, and sustained recovery was not achieved. In contrast, Case 2 showed evident and sustained symptom improvement following palliative-dose radiotherapy (20 Gy in 5 fractions). These contrasting clinical courses highlight that the reversibility of Horner syndrome depends more on the mechanism of sympathetic pathway involvement than on radiation dose alone. To explore potential factors influencing reversibility, we conducted a PubMed search of English-language case reports and case series using the index terms "Horner syndrome," "lung cancer," and "radiotherapy." Articles published up to December 2025 were screened for relevance to post-treatment neurological outcomes, and representative cases were summarized in Table 1 [4,5,7-11]. Across the reviewed cases, neurological improvement was reported predominantly in compression-dominant cases, whereas sustained recovery was uncommon when direct tumor invasion of the sympathetic pathway was considered the primary mechanism. Although the number of reported cases remains limited and heterogeneous, this pattern provides a consistent interpretive framework.

**Table 1:** Comparison of previously reported cases of malignancy-associated Horner syndrome with the present cases This table summarizes previously reported cases of Horner syndrome associated with malignant tumors, together with the two cases presented in this report. Cases are organized according to primary tumor, pattern of metastasis or local extension, presumed mode of sympathetic nerve involvement, treatment modality, and reversibility of Horner syndrome. The mode of sympathetic nerve involvement was categorized based on descriptions provided in each publication, including imaging findings and authors’ interpretations. Regarding the Horner triad, cases exhibiting all three features, i.e., miosis, ptosis, and facial anhidrosis, were classified as a complete triad, whereas cases exhibiting only some of these features were classified as incomplete. Reversibility of Horner syndrome was classified as improved when complete resolution or clear improvement was observed after treatment; transient improvement when improvement was temporary; partial improvement or incomplete improvement when improvement was limited; and no improvement when no apparent change was observed.

Author (Year)	Primary Tumor (Histological Type)	Metastatic/Extension Pattern	Mechanism of Sympathetic Involvement (as Described)	Horner Triad	Treatment	Reversibility of Horner Syndrome
Kovacic et al. (2007) [8]	Breast (invasive ductal carcinoma)	Pleural metastasis	Compression and/or invasion of the sympathetic chain due to pleural metastases	Complete	Chemotherapy	No improvement
Padungkiatsagul et al. (2018) [9]	Nasopharynx (undifferentiated non-keratinizing squamous cell carcinoma)	No apparent lymph node metastasis	Direct invasion of the primary tumor (skull base and pericarotid region)	Incomplete	Not described	Not described
Lele et al. (2019) [7]	Head and neck (squamous cell carcinoma) (n = 6)	Cervical lymph node metastasis	Encasement of the carotid sheath (compression ± invasion by lymph nodes or primary tumor)	Complete in most cases	Chemoradiotherapy ± surgery	Improvement in 1/6; no improvement in 5/6
Finsterer (2024) [5]	Lung (small cell carcinoma)	Mediastinal extension / superior vena cava compression	Venous congestion and compression of sympathetic fibers due to superior vena cava syndrome	Incomplete	Chemotherapy + superior vena cava stenting	Incomplete improvement
Akhtar et al. (2025) [10]	Thyroid (anaplastic thyroid carcinoma)	Cervical and mediastinal lymphadenopathy	Compression of the sympathetic pathway by the primary tumor	Incomplete	Molecular targeted therapy + surgery	Improvement
Hohs et al. (2025) [4]	Lung (carcinoma, histology not reported)	Supraclavicular lymph node metastasis	Compression of the sympathetic chain by supraclavicular lymph nodes	Incomplete	Chemotherapy + radiotherapy	Partial improvement
Mankal et al. (2025) [11]	Pleura (sarcomatoid malignant pleural mesothelioma)	Local extension	Compression of the upper thoracic sympathetic chain by the apical tumor	Complete	Supportive care / chemotherapy	No improvement
Present Case 1	Lung (adenocarcinoma)	No apparent metastasis	Presumed direct invasion of a perivascular primary tumor	Incomplete	Definitive chemoradiotherapy (60 Gy)	Transient improvement
Present Case 2	Lung (carcinoma, histology undetermined)	Supraclavicular lymph node metastasis	Presumed compression of the sympathetic chain by metastatic lymph nodes	Incomplete	Palliative radiotherapy (20 Gy)	Sustained improvement

In Case 1, the tumor circumferentially encased the right subclavian artery over more than 180 degrees, strongly suggesting direct involvement of sympathetic fibers within the perivascular region. In this context, the neural injury is most consistent with axonotmesis under the Seddon classification [12], corresponding to Sunderland Grade II or higher [13], reflecting established axonal damage. We postulate that the transient improvement observed at approximately 40 Gy resulted from a temporary reduction of compressive components due to tumor shrinkage. However, persistent axonal disruption likely limited full neurological recovery, leading to recurrence of ptosis after treatment completion. Radiotherapy-related fibrosis may have further impeded functional restoration.

In contrast, Case 2 was characterized primarily by compression from enlarged supraclavicular lymph nodes, without definitive imaging evidence of neural invasion. In this setting, the sympathetic pathway was likely affected predominantly by reversible functional impairment, corresponding to neurapraxia (Seddon) or Grade I injury (Sunderland). Relief of compression following lymph node shrinkage, together with reduction of perineural edema, likely restored microvascular perfusion and resolved conduction block within the sympathetic fibers. Because the structural integrity of the axons was presumably preserved, functional recovery became possible once the compressive and inflammatory factors were alleviated, resulting in sustained symptom improvement despite the relatively low radiation dose. However, detailed long-term neurological follow-up was limited, and additional imaging modalities such as MRI were not performed in the palliative context, representing a limitation of this report.

Taken together, our observations suggest that determining whether sympathetic dysfunction arises from reversible compression or irreversible structural neural damage is clinically more important than radiation dose in predicting neurological outcome. High-dose irradiation does not guarantee neurological recovery when structural injury predominates, whereas even palliative-dose radiotherapy may result in improvement when compression and edema are the dominant mechanisms. Careful pre-treatment imaging assessment may therefore help clinicians provide more accurate prognostic counseling and set realistic expectations for symptom recovery.

MR neurography has recently been proposed as a modality for grading peripheral nerve injury in accordance with the Sunderland classification [14]. Although not performed in our cases, future incorporation of such imaging techniques may allow more objective evaluation of sympathetic nerve injury. In advanced or palliative settings, however, the clinical utility of additional MRI must be balanced against patient burden and its impact on treatment decision-making.

This report has several important limitations. The conclusions are based on only two cases and a narrative review of previously published reports; therefore, the observations should be considered hypothesis-generating rather than definitive. Although based on limited data, our findings provide a mechanistic hypothesis that warrants further investigation. Prospective studies incorporating standardized imaging assessment and longitudinal neurological evaluation will be necessary to validate these preliminary observations. 

## Conclusions

In this article, we presented two cases of lung cancer-associated Horner syndrome demonstrating divergent clinical courses following radiotherapy. In the case in which pre-treatment imaging suggested direct sympathetic nerve invasion, neurological improvement was transient. In contrast, in the case considered to be predominantly caused by extrinsic compression from lymph node metastasis, sustained improvement was observed after radiotherapy. These observations suggest that post-treatment outcomes may vary according to the underlying mechanism of sympathetic nerve involvement. Estimation of the predominant mechanism, direct invasion versus compression, based on pre-treatment imaging findings may assist in anticipating the likelihood of neurological recovery after radiotherapy and in guiding clinical expectations. Although lung cancer-associated Horner syndrome is often regarded as having limited reversibility, these cases indicate that improvement may be achievable when compression is the dominant mechanism. However, given the limited sample size of two cases, these findings should be considered hypothesis-generating. Larger, systematic studies are required to validate these preliminary observations and to clarify the prognostic value of imaging-based mechanism assessment.
